# Supplementary material for: Coherent feedback leads to robust background compensation in oscillatory and non-oscillatory homeostats
Source: PLoS One. 2023 Aug 28;18(8):e0287083. doi: 10.1371/journal.pone.0287083 (PMC10461855; doi:10.1371/journal.pone.0287083)
Supplement: Attachment — Submitted filename: ref_PLOS_JUN23.pdf [file pone.0287083.s002.pdf]

Referee's report: PLOS ONE (PONE-D-23-16506)

Title: *"Coherent feedback leads to robust background compensation in oscillatory and non-oscillatory homeostats"*

Author: M. Nygard, P. Ruoff

Although the authors do a good job on the technical part of the manuscript (ms), which is more of an engineering analysis than a dynamic study, I cannot recommend the ms in its present form. The English scientific prose needs to be revised and improved. In general, the ms is awkward because the dynamic variables are never explicitly related to measurable physical properties of a biological system. The authors mention the photoadaptation of photoreceptors but never connect the dynamic variables (A, E, I_1, I_2) to the actual measurable properties of an experimental biological system. In summary, without a relationship between the dynamic variables in the ms, and real, measurable physical properties of a biological system, the ms is too vague to be of any interest to chemists, biologists, and physiologists. I would ask the authors, what is your target audience?

Although I could guess that the variables (I_1, I_2) are some kind of feedback inputs, I could not understand the dynamic variables 'a' and 'e,' which the authors do not mention in their ms. What is the difference between e, in Eq. 4, 12, and a, in Eqs. 20, 28? The physical interpretation of these dynamic variables is not explained in the ms. In line 20, the authors try to explain the onset of oscillations. But why is it that the authors are not considering bifurcation diagrams to find, in particular, the Poincare-Andronov-Hopf (PAH) bifurcation? Bifurcation diagrams complement and determine the stable and unstable branches of the steady states of dynamic systems.

In line 258, the authors try to relate the Hill equation to the Michaelis-Menten equation.

But the Michaelis-Menten is a particular case of the Hill equation with $\alpha = 1$, which is a more general equation. In the ms, one could interpret the generality of the Hill equation incorrectly. But more importantly, if the authors use Michaelis-Mente expressions, one may wonder if the authors are considering enzymatic reactions. If they are, the authors could give the readers some examples.

Here are some issues:

1 - Besides the issue of the ms belonging to an engineering journal, the revised ms does not, in my opinion, reaches an appropriate quality of the written modern scientific English prose.

2 - The authors ignored punctuation rules and missed too many commas. The ms should be revised for punctuation.

3 - Quotates should be revised and corrected. For example, in line 76, 'outer' should be 'outer.' Notice the first quotation

4 - One cannot start a sentence with an abbreviation, like Fig 4a, or a symbol like E.

5 - in modern scientific English prose, one must write using complete sentences. In particular, all the figure captions should be written with complete sentences and should start with " Figure ...," rather than "Fig ...".

6 - In general, the ms is written in awkward English prose. A knowledgeable person of written English prose should revise the ms.

7 - In line 60, the sentence " In the below calculations we have used .." is missing the proper punctuation and is too awkward and should be rewritten.

8 In line 71, another awkward sentence, "The type of oscillations we here focus on ..".

9 - in modern scientific English prose, one must avoid short paragraphs, in particular one-sentence paragraphs, like in lines 20 and 125.

10 - In a paragraph starting at line 281, one should not quote other authors in an original research report. Authors should leave those quotations to review or historically relevant reports.

In modern scientific English prose, one writes in an active voice. The use of passive voice is restricted to cases where one does not know the subject or does not care about the subject. For example, in Figures 1 and 2, the authors write " flashes were applied". The natural question is who applied these flashes. It is unclear if the authors or other researchers gathered the information in Figures 1 and 2. Also notice line 27, where the authors write "...control was previously observed by us .." The sentence is awkward and should be written in an active voice.

The ms lacks a relevant Conclusion section, where the authors should compare and relate their results with at least one physical system. In other words, if the authors are interested in retinal light adaptation, they should connect the dynamic variables (A, E, I_1, I_2, e or a) to the measurable physical properties of a biological system.

Although I find the technical aspects developed by the authors publishable in an **engineering** journal, I do not recommend publication in PLOS ONE in its present form. Still, I strongly recommend thoroughly revising the ms for clarity.
