# Supplementary material for: Coherent feedback leads to robust background compensation in oscillatory and non-oscillatory homeostats
Source: PLoS One. 2023 Aug 28;18(8):e0287083. doi: 10.1371/journal.pone.0287083 (PMC10461855; doi:10.1371/journal.pone.0287083)
Supplement: Attachment — Submitted filename: Response to Reviewers.pdf [file pone.0287083.s003.pdf]

Changes made in the revised manuscript based on the comments by Reviewer #1

Changes made in the manuscript with respect to the comments by Reviewer #1 are indicated in red in the marked-up copy of the revised manuscript.

Reviewer #1: The manuscript titled 'Coherent feedback leads to robust background compensation in oscillatory and nonoscillatory homeostats' by Nygård et al. proposes a control scheme that can provide disturbance rejections in the presence of multi-dimensional disturbances. The proposed scheme is technically rigorous and has been corroborated with sufficient case studies, thereby containing the potential to be a valuable addition to the literature. However, the following concerns may be addressed before the publication of this manuscript. Further, all the required details for the simulation studies have been made available.
Response: *We would like to thank the reviewer for the positive assessment.*

Reviewer #1: 1. In the very first sentence of the abstract, the authors need to explicitly mention that the step perturbation is applied at the output terminal and, further, the sign of the step perturbation.
Response: *In the first sentence of the abstract we have actually mentioned that a step perturbation is applied to the controlled variable A. Instead of using 'output terminal', we would like to keep the term 'controlled variable' for A, and ΔA for the response amplitude. Since we focus on reaction kinetics and concentrations (which are positive entities) we have now explicitly added in the abstract that we look at concentrations of the controlled variable A. Furthermore, to indicate that perturbation and background 'pull in the same direction', we have added in the abstract that a step perturbation is applied besides a constant background. We hope that this will give the reader a first correct association of what a background is.*

Reviewer #1: 2. The term 'background' in the context of regulatory control would not be very clear to a control theorist. Therefore, the authors may consider spending a few lines on clarifying what changing background means in the language of systems theory. As I understand, the background in this manuscript, is mapped to the output of the control (also known as the error variable in control literature). Therefore, at the onset of step perturbations, in the absence of additional supervisory control, the existing integral control scheme can not drive the output to the previous steady state due to the change in the control output of the controller - This needs to be reflected properly in the manuscript.
Response: *To clarify the background concept we rewrote the subsection of integral control, which now includes the definition of a background. Please, see section 'Integral control, step perturbations and the background concept' under 'Materials and methods'. Fig 3 has also been slightly changed. In panel b we now explicitly indicate that k_1 represents a step perturbation, while k_2, related to the background reaction, is kept constant. As the reviewer correctly points out, in the absence of additional supervisory control, different but constant backgrounds will show different steady states, while the controlled variable is still kept at its set-point. This is reflected in Fig 1 by the diminished response amplitudes V_i when constant background light levels are increased. Motif 2 has been found to describe the situation of the diminishing response amplitudes at different backgrounds (Fig 1) quite well (for details, see Ref 5). With reference to the changed response kinetics at different backgrounds (Fig 1), we mention the focus of the present paper, i.e. the novel finding of conditions where the response kinetics of the controlled variable becomes independent of the background. Please, see lines 52-71 and revised Fig 3 in the marked-up copy. In addition, we further discuss the background concept in the section 'Is retinal light adaptation background compensated?' at the end of the manuscript (see lines 272-280).*

Reviewer #1: 3. Is it not already well-established in the literature of regulator theory that an integral control scheme is robust to parametric fluctuations as long as 1) the fluctuated parameters belong to the process and 2)

the overall stability is not altered?. Further, this has also been shown to be the case by Bhattacharya et al. (2023), Mathematical biosciences. The K10 or the background species, only appears at the process dynamics. Therefore, any perturbations on K1 should ideally have no effect in dictating the steady state of the system. Therefore, I request the authors to clarify and rephrase the contributions of this manuscript in light of this observation.
Response: *Thank you for making us aware about the work by Bhattacharya et al. based on linearized mathematical models, which we have now cited. In addition, we have also cited the approach by Ponsiglione et al. based on generic nonlinear mathematical models. Please, note that these more mathematically inclined methods differ from our approach here, which is based on mass action kinetics. See lines 81-84 in the marked-up copy describing these aspects.*
We agree mostly which what the reviewer says about integral control, ***except*** *from the statement:*
'The K10 or the background species, only appears at the process dynamics. Therefore, any perturbations on K1 should ideally have no effect in dictating the steady state of the system.'
This general statement about the steady state of an integral-controlled system is not entirely correct, because the controller (manipulated) variables (here E, I_1, or I_2) have to change their steady states in order to move the controlled variable to its set-point. As an outcome of this, changed backgrounds have a clear influence on the <u>*response kinetics*</u> *of integral controllers, as indicated in Fig 1 and in the work by Grini et al. (Ref 5). With respect to the contribution of this manuscript, we refer to lines 66-70 in the marked-up copy:*
'In fact, motif 2 has been found to describe light adaptations and the influence of different background light intensities, as shown in Fig 1, relatively well [5]. In this paper we describe novel findings how a background can be compensated for, such that the response kinetics become independent of different but constant backgrounds.'

Reviewer #1: 4. A large part of the manuscript assumes zero-order kinetics. While I appreciate the fact that the zeroth-order kinetics is mathematically convenient both for analysis and demonstration, it also limits the generalizability of the results to a great extent. Therefore, at least a brief discussion of possible generalizations to other rate kinetics is warranted.
Response: *We had included the following paragraph in the manuscript to mention other approaches to include integral control in the marked-up copy:*

Line 71: ***There are several kinetic requirements which can lead to integral control.***

Lines 74-80: ***In the following calculations we have used zero-order kinetics to implement integral control [17,22-24]. However, it should be mentioned that there are other kinetics conditions to achieve integral control, such as antithetic control [21,25,26], which often will show identical resetting behaviors as in zero-order control [5,26]. Also, autocatalytic reactions can be used to obtain integral control [27-29], which generally will show much faster resetting kinetics in comparison when integral control is introduced by zero-order kinetics [30,31].***

Together with mentioning the approaches by Bhattacharya et al. (Refs 32,33) and Ponsiglione et al. (Ref 34) in addition to Refs 17-21, 24-25, and 27, we feel we pointed to other approaches to achieve robust/integral control.

Finally, we would also like to mention that zero-order kinetics is not only mathematically convenient, but has a physical-chemical foundation in catalyzed reaction systems (see for example, Ref 35).

Reviewer #1: Minor: Please perform a thorough reading of the manuscript. Although the manuscript is well-written, and the language is quite good at most of the time, I can spot multiple proofing errors throughout.
Response: *Thank you. We have carefully proof-read the revised version and made additional corrections outlined in red in the marked-up copy.*

Changes made in the revised manuscript based on the comments by Reviewer #2

Changes made in the manuscript with respect to the comments by Reviewer #1 are indicated in blue in the marked-up copy of the revised manuscript.

Reviewer #2: Although the authors do a good job on the technical part of the manuscript (ms), which is more of an engineering analysis than a dynamic study, I cannot recommend the ms in its present form. The English scientific prose needs to be revised and improved. In general, the ms is awkward because the dynamic variables are never explicitly related to measurable physical properties of a biological system. The authors mention the photoadaptation of photoreceptors but never connect the dynamic variables (A, E, I_1, I_2) to the actual measurable properties of an experimental biological system. In summary, without a relationship between the dynamic variables in the ms, and real, measurable physical properties of a biological system, the ms is too vague to be of any interest to chemists, biologists, and physiologists. I would ask the authors, what is your target audience?
Response: *Our manuscript describes reaction kinetic systems with integral control in which the contribution of backgrounds, as defined in the manuscript, are compensated for. Related kinetic systems have been described by us before in PLOS ONE, for example the scheme in Fig 4a with respect to robust frequency control (see Ref 11). As our models are based on reaction kinetics, the described systems are of a potentially physical nature, and we therefore think that the results described here can be of interest to chemists, (synthetic) biologist, control engineers interested in the regulation of biological and chemical systems, as well as mathematicians.*

Reviewer #2: Although I could guess that the variables (I_1, I_2) are some kind of feedback inputs, I could not understand the dynamic variables 'a' and 'e,' which the authors do not mention in their ms. What is the difference between e, in Eq. 4, 12, and a, in Eqs. 20, 28? The physical interpretation of these dynamic variables is not explained in the ms. In line 20, the authors try to explain the onset of oscillations. But why is it that the authors are not considering bifurcation diagrams to find, in particular, the Poincare-Andronov-Hopf (PAH) bifurcation? Bifurcation diagrams complement and determine the stable and unstable branches of the steady states of dynamic systems.
Response: *Compounds a and e are precursors to respectively A and E, which were included in a previous study of these networks (see Ref 11). In Ref 11 the inclusion of such precursors were found to promote limit-cycle oscillations. To address this explicitly we have added in the legend of Fig 4 the following sentence: 'The addition of precursor e was found [11] to promote limit-cycle oscillations.'*
With respect to the generation of oscillations, the reviewer asks why we do not consider bifurcation analysis, in particular the Hopf bifurcation, also called the Poincare-Andronov-Hopf (PAH) bifurcation. The reason for this is that we are primarily interested in the (kinetic) criteria which promote oscillations within a negative feedback loop. In Ref 11 we found that zero-order kinetics in the removal of controlled and manipulated variables promote oscillations, as there is a relationship between these zero-order systems and underlying core quasi-harmonic and quasi-conservative oscillators. Bifurcation analysis is -in our view- slightly different, because you already need to have knowledge of a certain nonoscillatory-oscillatory transition with respect to a given bifurcation parameter. Although bifurcation theory is of interest to characterize such transitions, it is less interesting to us, as we want to get our hands on kinetic conditions how oscillation in a negative feedback can arise.

Reviewer #2: In line 258, the authors try to relate the Hill equation to the Michaelis-Menten equation. But the Michaelis-Menten is a particular case of the Hill equation with $\alpha = 1$, which is a more general equation. In the ms, one could interpret the generality of the Hill equation incorrectly. But more importantly, if the authors use Michaelis-Mente expressions, one may wonder if the authors are considering enzymatic reactions. If they are, the authors could give the readers some examples.
Response: *Yes, we agree that the Michaelis-Menten equation is a special case of the Hill equation with*

cooperativity α=1. To avoid possible confusion, we have rewritten that part and call Eq 32 as 'Hill equation' (see lines 282-283 in marked-up copy).
As described in the text, Eq 32 was taken from the description/review by Dowling (Ref 3). There, Eq 32 was used as a generic model and has been analyzed with respect to different light-responsive cells in the vertebrate retina. No specific enzymes have been studied. Since photoreceptor cells have been found to have α=1, we further mention that the Hill equation is changed in that case into a Michaelis-Menten equation (see lines 284-285).

Issues:
Reviewer #2: 1 - Besides the issue of the ms belonging to an engineering journal, the **revised ms** does not, in my opinion, reaches an appropriate quality of the written modern scientific English prose.
Response: *We were quite surprised by the utterly negative description of our English. We normally get the response that our English is reasonably good. As Reviewer #1 has put it: '...the manuscript is well-written, and the language is quite good...'. However, we have carefully gone through our manuscript and made changes there where we thought the style could be improved (see changes outlined in red in the marked-up copy of the manuscript).*
We wonder why the reviewer refers to a 'revised ms' (indicated in bold above). Our manuscript has been an initial and not a revised submission to PLOS ONE.
We oppose the reviewer's suggestion that our manuscript should be sent to another more engineering related journal. Our studies combine reaction kinetics with control-engineering methods. PLOS ONE is an interdisciplinary broad scope journal, where we wish to publish our results. We have previously published in PLOS ONE on related subjects, which combine reaction kinetics with control-engineering methods.

Reviewer #2: 2 - The authors ignored punctuation rules and missed too many commas. The ms should be revised for punctuation.
Response: *We have carefully proof-read our manuscript.*

Reviewer #2: 3 - Quotates should be revised and corrected. For example, in line 76, 'outer' should be 'outer.' Notice the first quotation
Response: *Thank you for spotting this. We have corrected the manuscript with respect to quotations.*

Reviewer #2: 4 - One cannot start a sentence with an abbreviation, like Fig 4a, or a symbol like E.
5 - in modern scientific English prose, one must write using complete sentences. In particular, all the figure captions should be written with complete sentences and should start with " Figure ...," rather than "Fig ...".
Response: *Please, be aware that the manuscript has undergone a screening by the editorial staff of PLOS ONE to meet the stylistic requirements of the journal. We have used a LaTeX template, where it is explicitly stated:*

% For figure citations, please use "Fig" instead of "Figure".
% Use "Eq" instead of "Equation" for equation citations.

The template automatically uses 'Fig' instead of 'Figure' in the figure legend.

Reviewer #2: 6 - In general, the ms is written in awkward English prose. A knowledgeable person of written English prose should revise the ms.
Response: *Please, see our responses to points 1 and 2 above.*

Reviewer #2: 7 - In line 60, the sentence " In the below calculations we have used .." is missing the proper punctuation and is too awkward and should be rewritten.
Response: *We don't see anything wrong with this sentence, but have changed 'below' with 'following' (line 74 in marked-up copy).*

Reviewer #2: 8 In line 71, another awkward sentence, "The type of oscillations we here focus on ..".
Response: *We have rewritten the sentence to (see lines 90-92):*
'The here considered oscillators show frequency homeostasis based on a two-layered negative feedback structure.'

Reviewer #2: 9 - in modern scientific English prose, one must avoid short paragraphs, in particular one-sentence paragraphs, like in lines 20 and 125.
Response: *We have now removed the one sentence paragraphs (see lines 19 and 146).*

Reviewer #2: 10 - In a paragraph starting at line 281, one should not quote other authors in an original research report. Authors should leave those quotations to review or historically relevant reports.
Response: *We don't see any reason why one should not quote an author in a research paper, especially when trying to review and making more clear the other author's position/opinion. However, we have now paraphrased our citation of Dowling's text. Please, see lines 307-311.*

Reviewer #2: In modern scientific English prose, one writes in an active voice. The use of passive voice is restricted to cases where one does not know the subject or does not care about the subject. For example, in Figures 1 and 2, the authors write " flashes were applied". The natural question is who applied these flashes. It is unclear if the authors or other researchers gathered the information in Figures 1 and 2. Also notice line 27, where the authors write "...control was previously observed by us .." The sentence is awkward and should be written in an active voice.
Response: *In both legends of Fig 1 and Fig 2 we have explicitly stated that these figures were redrawn and we cited the sources of the original figures.*
With respect to the sentence starting at line 26 in the original manuscript, we have rewritten the sentence to (starting line 28):
'Although we previously observed robust frequency control [11], we did not recognize at that time the background-compensating property of coherent feedback.'

Reviewer #2: The ms lacks a relevant Conclusion section, where the authors should compare and relate their results with at least one physical system. In other words, if the authors are interested in retinal light adaptation, they should connect the dynamic variables (A, E, I_1, I_2, e or a) to the measurable physical properties of a biological system.
Response: *We think that our 'Conclusion and outlook' section, starting line 318, is relevant and describes the significance of our findings. Our paper is <u>not</u> about retinal light adaptation (for that see Ref 5), but about discussing and clearing up the term 'background compensation', which was used somewhat sloppy in the literature. The manuscript further describes novel regulatory conditions which actually can lead to robust background compensation.*

Reviewer #2: Although I find the technical aspects developed by the authors publishable in an **engineering** journal, I do not recommend publication in PLOS ONE in its present form. Still, I strongly recommend thoroughly revising the ms for clarity.

Response: *As indicated above, we think that PLOS ONE is a good choice to publish our findings. It is described as an interdisciplinary broad scope journal. We have carefully revised the manuscript.*

Additional changes made in the revised manuscript

- We have made minor rewordings, which are indicated in red in the marked-up copy.

- In Eq 18 we discovered an error, where the removal of A has been written in form of saturation kinetics with respect to A. The correct term is '$-k_g \cdot A \cdot I_1$' (outlined in purple in the marked-up copy), i.e. the degradation/removal of A by I_1 is first-order with respect to A.
