# Supplementary material for: Coherent feedback leads to robust background compensation in oscillatory and non-oscillatory homeostats
Source: PLoS One. 2023 Aug 28;18(8):e0287083. doi: 10.1371/journal.pone.0287083 (PMC10461855; doi:10.1371/journal.pone.0287083)
Supplement: Peer review history

# Peer review: Decision Letter 0

**DOI:** 10.1371/journal.pone.0287083.r001

**Authors:** Karthik Raman

12 Jul 2023

PONE-D-23-16506Coherent feedback leads to robust background compensation in oscillatory and non-oscillatory homeostatsPLOS ONE

Dear Dr. Ruoff,

Thank you for submitting your manuscript to PLOS ONE. After careful consideration, we feel that it has merit but does not fully meet PLOS ONE’s publication criteria as it currently stands. Therefore, we invite you to submit a revised version of the manuscript that addresses the points raised during the review process.

Please submit your revised manuscript by Aug 26 2023 11:59PM. If you will need more time than this to complete your revisions, please reply to this message or contact the journal office at plosone@plos.org. When you're ready to submit your revision, log on to https://www.editorialmanager.com/pone/ and select the 'Submissions Needing Revision' folder to locate your manuscript file.

Please include the following items when submitting your revised manuscript:

- A rebuttal letter that responds to each point raised by the academic editor and reviewer(s). You should upload this letter as a separate file labeled 'Response to Reviewers'.
- A marked-up copy of your manuscript that highlights changes made to the original version. You should upload this as a separate file labeled 'Revised Manuscript with Track Changes'.
- An unmarked version of your revised paper without tracked changes. You should upload this as a separate file labeled 'Manuscript'.

If you would like to make changes to your financial disclosure, please include your updated statement in your cover letter. Guidelines for resubmitting your figure files are available below the reviewer comments at the end of this letter.

If applicable, we recommend that you deposit your laboratory protocols in protocols.io to enhance the reproducibility of your results. Protocols.io assigns your protocol its own identifier (DOI) so that it can be cited independently in the future. For instructions see: https://journals.plos.org/plosone/s/submission-guidelines#loc-laboratory-protocols. Additionally, PLOS ONE offers an option for publishing peer-reviewed Lab Protocol articles, which describe protocols hosted on protocols.io. Read more information on sharing protocols at https://plos.org/protocols?utm_medium=editorial-email&utm_source=authorletters&utm_campaign=protocols.

We look forward to receiving your revised manuscript.

Kind regards,

Karthik Raman, Ph.D.

Academic Editor

PLOS ONE

Journal Requirements:

When submitting your revision, we need you to address these additional requirements.

1. Please ensure that your manuscript meets PLOS ONE's style requirements, including those for file naming. The PLOS ONE style templates can be found at 

https://journals.plos.org/plosone/s/file?id=wjVg/PLOSOne_formatting_sample_main_body.pdf and 

https://journals.plos.org/plosone/s/file?id=ba62/PLOSOne_formatting_sample_title_authors_affiliations.pdf

**Additional Editor Comments:**

Please pay specific attention to the detailed comments of both reviewers.

[Note: HTML markup is below. Please do not edit.]

Reviewers' comments:

Reviewer's Responses to Questions

**Comments to the Author**

1. Is the manuscript technically sound, and do the data support the conclusions?

The manuscript must describe a technically sound piece of scientific research with data that supports the conclusions. Experiments must have been conducted rigorously, with appropriate controls, replication, and sample sizes. The conclusions must be drawn appropriately based on the data presented. 

Reviewer #1: Yes

Reviewer #2: Partly

**********

2. Has the statistical analysis been performed appropriately and rigorously? 

Reviewer #1: N/A

Reviewer #2: N/A

**********

3. Have the authors made all data underlying the findings in their manuscript fully available?

The PLOS Data policy requires authors to make all data underlying the findings described in their manuscript fully available without restriction, with rare exception (please refer to the Data Availability Statement in the manuscript PDF file). The data should be provided as part of the manuscript or its supporting information, or deposited to a public repository. For example, in addition to summary statistics, the data points behind means, medians and variance measures should be available. If there are restrictions on publicly sharing data—e.g. participant privacy or use of data from a third party—those must be specified.

Reviewer #1: Yes

Reviewer #2: Yes

**********

4. Is the manuscript presented in an intelligible fashion and written in standard English?

PLOS ONE does not copyedit accepted manuscripts, so the language in submitted articles must be clear, correct, and unambiguous. Any typographical or grammatical errors should be corrected at revision, so please note any specific errors here.

Reviewer #1: Yes

Reviewer #2: No

**********

5. Review Comments to the Author

Please use the space provided to explain your answers to the questions above. You may also include additional comments for the author, including concerns about dual publication, research ethics, or publication ethics. (Please upload your review as an attachment if it exceeds 20,000 characters)

Reviewer #1: The manuscript titled ‘Coherent feedback leads to robust background compensation in oscillatory and nonoscillatory homeostats’ by Nyg˚ard et al. proposes a control scheme that can provide disturbance rejections in the presence of multi-dimensional disturbances. The proposed scheme is technically rigorous and has been corroborated with sufficient case studies, thereby containing the potential to be a valuable addition to the literature. However, the following concerns may be addressed before the publication of this manuscript. Further, all the required details for the simulation studies have been made available.

 1. In the very first sentence of the abstract, the authors need to explicitly mention that the step perturbation is applied at the output terminal and, further, the sign of the step perturbation.

2. The term ‘background’ in the context of regulatory control would not be very clear to a control theorist. Therefore, the authors may consider spending a few lines on clarifying what changing background means in the language of systems theory. As I understand, the background in this manuscript, is mapped to the output of the control (also known as the error variable in control literature). Therefore, at the onset of step perturbations, in the absence of additional supervisory control, the existing integral control scheme can not drive the output to the previous steady state due to the change in the control output of the controller— This needs to be reflected properly in the manuscript.

3. Is it not already well-established in the literature of regulator theory that an integral control scheme is robust to parametric fluctuations as long as 1) the fluctuated parameters belong to the process and 2) the overall stability is not altered?. Further, this has also been shown to be the case by Bhattacharya et al. (2023), Mathematical biosciences. The K10 or the background species, only appears at the process dynamics. Therefore, any perturbations on K1 should ideally have no effect in dictating the steady state of the system. Therefore, I request the authors to clarify and rephrase the contributions of this manuscript in light of this observation.

4. A large part of the manuscript assumes zero-order kinetics. While I appreciate the fact that the zeroth-order kinetics is mathematically convenient both for analysis and demonstration, it also limits the generalizability of the results to a great extent. Therefore, at least a brief discussion of possible generalizations to other rate kinetics is warranted.

Minor: Please perform a thorough reading of the manuscript. Although the manuscript is well-written, and the language is quite good at most of the time, I can spot multiple proofing errors throughout.

Reviewer #2: Referee's report: PLOS ONE (PONE-D-23-16506)\\\\

Title:{\\it ``Coherent feedback leads to robust background compensation in oscillatory and non-oscillatory homeostats'' }\\\\

Author: M. Nygard, P. Ruoff\\\\

Although the authors do a good job on the technical part of the manuscript (ms), which is more of an engineering analysis than a dynamic study, I cannot recommend the ms in its present form. The English scientific prose needs to be revised and improved. In general, the ms is awkward because the dynamic variables are never explicitly related to measurable physical properties of a biological system. The authors mention the photoadaptation of photoreceptors but never connect the dynamic variables ($A, E, I_1, I_2$) to the actual measurable properties of an experimental biological system. In summary, without a relationship between the dynamic variables in the ms, and real, measurable physical properties of a biological system, the ms is too vague to be of any interest to chemists, biologists, and physiologists. I would ask the authors, what is your target audience?

Although I could guess that the variables ($I_1, I_2$) are some kind of feedback inputs, I could not understand the dynamic variables `a' and `e,' which the authors do not mention in their ms. What is the difference between e, in Eq. 4, 12, and a, in Eqs. 20, 28? The physical interpretation of these dynamic variables is not explained in the ms.

In line 20, the authors try to explain the onset of oscillations. But why is it that the authors are not considering bifurcation diagrams to find, in particular, the Poincare-Andronov-Hopf (PAH) bifurcation? Bifurcation diagrams complement and determine the stable and unstable branches of the steady states of dynamic systems.

In line 258, the authors try to relate the Hill equation to the Michaelis-Menten equation. But the Michaelis-Menten is a particular case of the Hill equation with $\\alpha =1$, which is a more general equation. In the ms, one could interpret the generality of the Hill equation incorrectly. But more importantly, if the authors use Michaelis-Mente expressions, one may wonder if the authors are considering enzymatic reactions. If they are, the authors could give the readers some examples.

Here are some issues:\\\\

1 - Besides the issue of the ms belonging to an engineering journal, the revised ms does not, in my opinion, reaches an appropriate quality of the written modern scientific English prose.\\\\

2 - The authors ignored punctuation rules and missed too many commas. The ms should be revised for punctuation.\\\\

3 - Quotates should be revised and corrected. For example, in line 76, 'outer' should be `outer.' Notice the first quotation\\\\

4 - One cannot start a sentence with an abbreviation, like Fig 4a, or a symbol like E.\\\\

5 - in modern scientific English prose, one must write using complete sentences. In particular, all the figure captions should be written with complete sentences and should start with `` Figure ...,'' rather than ``Fig ...''.\\\\

6 - In general, the ms is written in awkward English prose. A knowledgeable person of written English prose should revise the ms.\\\\

7 - In line 60, the sentence `` In the below calculations we have used ..'' is missing the proper punctuation and is too awkward and should be rewritten.\\\\

8 In line 71, another awkward sentence, ``The type of oscillations we here focus on ..''.\\\\

9 - in modern scientific English prose, one must avoid short paragraphs, in particular one-sentence paragraphs, like in lines 20 and 125.\\\\

10 - In a paragraph starting at line 281, one should not quote other authors in an original research report. Authors should leave those quotations to review or historically relevant reports.\\\\

In modern scientific English prose, one writes in an active voice. The use of passive voice is restricted to cases where one does not know the subject or does not care about the subject. For example, in Figures 1 and 2, the authors write `` flashes were applied''. The natural question is who applied these flashes. It is unclear if the authors or other researchers gathered the information in Figures 1 and 2. Also notice line 27, where the authors write ``...control was previously observed by us ..'' The sentence is awkward and should be written in an active voice.

The ms lacks a relevant Conclusion section, where the authors should compare and relate their results with at least one physical system. In other words, if the authors are interested in retinal light adaptation, they should connect the dynamic variables $(A, E, I_1, I_2, e$ or $a$) to the measurable physical properties of a biological system.

Although I find the technical aspects developed by the authors publishable in an {\\bf engineering} journal, I do not recommend publication in PLOS ONE in its present form. Still, I strongly recommend thoroughly revising the ms for clarity.

**********

6. PLOS authors have the option to publish the peer review history of their article (what does this mean?). If published, this will include your full peer review and any attached files.

If you choose “no”, your identity will remain anonymous but your review may still be made public.

**Do you want your identity to be public for this peer review?** For information about this choice, including consent withdrawal, please see our Privacy Policy.

Reviewer #1: No

Reviewer #2: No

**********

[NOTE: If reviewer comments were submitted as an attachment file, they will be attached to this email and accessible via the submission site. Please log into your account, locate the manuscript record, and check for the action link "View Attachments". If this link does not appear, there are no attachment files.]

While revising your submission, please upload your figure files to the Preflight Analysis and Conversion Engine (PACE) digital diagnostic tool, https://pacev2.apexcovantage.com/. PACE helps ensure that figures meet PLOS requirements. To use PACE, you must first register as a user. Registration is free. Then, login and navigate to the UPLOAD tab, where you will find detailed instructions on how to use the tool. If you encounter any issues or have any questions when using PACE, please email PLOS at figures@plos.org. Please note that Supporting Information files do not need this step.

Attachment Submitted filename: ref_PLOS_JUN23.pdf Click here for additional data file.

# Author comment: Author response to Decision Letter 0

**DOI:** 10.1371/journal.pone.0287083.r002

10 Aug 2023

Please, see the attached rebuttal letter.

Attachment Submitted filename: Response to Reviewers.pdf Click here for additional data file.

# Editor report: Decision Letter 1

**DOI:** 10.1371/journal.pone.0287083.r003

**Authors:** Karthik Raman

15 Aug 2023

Coherent feedback leads to robust background compensation in oscillatory and non-oscillatory homeostats

PONE-D-23-16506R1

Dear Dr. Ruoff,

We’re pleased to inform you that your manuscript has been judged scientifically suitable for publication and will be formally accepted for publication once it meets all outstanding technical requirements.

Within one week, you’ll receive an e-mail detailing the required amendments. When these have been addressed, you’ll receive a formal acceptance letter and your manuscript will be scheduled for publication.

An invoice for payment will follow shortly after the formal acceptance. To ensure an efficient process, please log into Editorial Manager at http://www.editorialmanager.com/pone/, click the 'Update My Information' link at the top of the page, and double check that your user information is up-to-date. If you have any billing related questions, please contact our Author Billing department directly at authorbilling@plos.org.

If your institution or institutions have a press office, please notify them about your upcoming paper to help maximize its impact. If they’ll be preparing press materials, please inform our press team as soon as possible -- no later than 48 hours after receiving the formal acceptance. Your manuscript will remain under strict press embargo until 2 pm Eastern Time on the date of publication. For more information, please contact onepress@plos.org.

Kind regards,

Karthik Raman, Ph.D.

Academic Editor

PLOS ONE

Additional Editor Comments (optional):

The authors have satisfactorily addressed all reviewer comments.

# Editor report: Acceptance letter

**DOI:** 10.1371/journal.pone.0287083.r004

**Authors:** Karthik Raman

17 Aug 2023

PONE-D-23-16506R1 

Coherent feedback leads to robust background compensation in oscillatory and non-oscillatory homeostats 

Dear Dr. Ruoff:

I'm pleased to inform you that your manuscript has been deemed suitable for publication in PLOS ONE. Congratulations! Your manuscript is now with our production department. 

If your institution or institutions have a press office, please let them know about your upcoming paper now to help maximize its impact. If they'll be preparing press materials, please inform our press team within the next 48 hours. Your manuscript will remain under strict press embargo until 2 pm Eastern Time on the date of publication. For more information please contact onepress@plos.org.

If we can help with anything else, please email us at plosone@plos.org. 

Thank you for submitting your work to PLOS ONE and supporting open access. 

Kind regards, 

PLOS ONE Editorial Office Staff

on behalf of

Dr. Karthik Raman 

Academic Editor

PLOS ONE
